# Primary hepatic lymphoma in a patient with cirrhosis: a case report

**DOI:** 10.1186/s13256-020-02471-0

**Published:** 2020-09-26

**Authors:** Eduardo Dantas, Joana Santos, Mariana Coelho, Cristiana Sequeira, Inês Santos, Cláudia Cardoso, Ana Paula Oliveira

**Affiliations:** 1 Gastroenterology Department, Centro Hospitalar de Setúbal, Setúbal, Portugal; 2 Oncology Department, Centro Hospitalar de Setúbal, Setúbal, Portugal

**Keywords:** Primary hepatic lymphoma, Diffuse large B-cell lymphoma

## Abstract

**Background:**

Primary hepatic lymphoma is a very uncommon disease. Due to its nonspecific clinical, laboratory, and imaging findings, it is often misdiagnosed. Liver biopsy is required to make a final diagnosis. Chemotherapy is the current gold standard of treatment.

**Case presentation:**

An asymptomatic 65-year-old Caucasian man with Child-Pugh class A cirrhosis presented to our hospital with a nodular lesion seen on a routine surveillance abdominal ultrasound. His physical examination revealed hepatomegaly and no other significant findings. Magnetic resonance imaging of the abdomen showed a voluminous nodule on the left lobe with heterogeneous contrast enhancement. His liver biopsy was compatible with diffuse large B-cell lymphoma. Systemic staging showed no evidence of nodal or bone marrow involvement, confirming the diagnosis of primary hepatic lymphoma. He was treated with chemotherapy. However, he developed febrile neutropenia after one of the cycles and died.

**Conclusions:**

In this article, we report a rare presentation of non-Hodgkin lymphoma and review the current literature on clinical features, diagnosis, and management.

## Introduction

Diffuse large B-cell lymphoma (DLBCL) is the most common histological type of non-Hodgkin lymphoma (NHL), being responsible for nearly 30% of NHL cases [1], with an annual incidence in Europe of 3.8 per 100,000 [2]. Even though the liver contains lymphoid tissue, host factors may make it a poor environment for the development of malignant lymphoma. More often, NHL affects the liver in advanced stages of a systemic disease and rarely as a primary hepatic lymphoma (PHL) [3]. PHL accounts for 0.4% of extranodal NHL and 0.016% of all NHL [4]. The majority of PHLs are B-cell lymphomas, with DLBCL being the most common subtype [5]. Due to its rare occurrence, the pathogenesis of PHL is still unclear. Various etiologic factors have been proposed, including viral infections such as hepatitis B virus (HBV), hepatitis C virus (HCV), human immunodeficiency virus (HIV), and Epstein-Barr virus (EBV), as well as liver cirrhosis, autoimmune disorders, and immunosuppressive therapy, although risk rates in these patients have not been reported so far due to disease rarity [6]. Chronic liver disease before the onset of PHL has been reported in 9.6% of 52 patients in a small case series in Western countries [7]. HCV infection is found in 20–60% of patients, indicating that it may be involved in the pathogenesis of the disease [8]. Clinical manifestations, laboratory findings, and imaging features are usually nonspecific, making it difficult to distinguish from more common neoplastic entities, such as primary liver cancer or metastatic disease.

## Case presentation

We present a case of a 65-year-old Caucasian man with Child-Pugh class A alcoholic cirrhosis diagnosed after a hospitalization due to esophageal variceal bleeding and portal hypertensive gastropathy. Since then, he had been seen in follow-up in a hepatology clinic and was prescribed propranolol 10 mg four times daily and advised to initiate alcohol withdrawal.

**Fig. 1:**
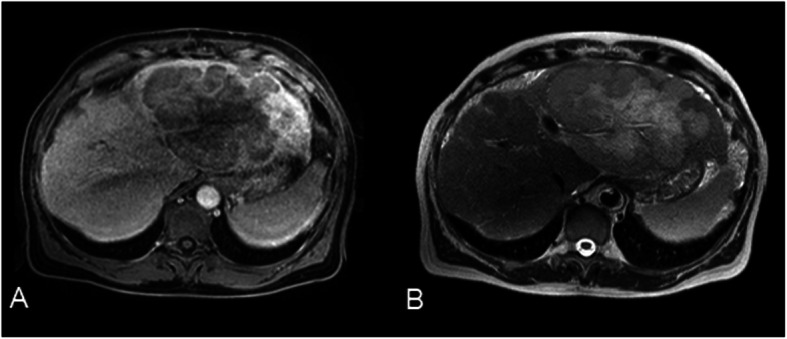
Abdominal magnetic resonance imaging revealing a nodular lesion in the left lobe measuring 15.6 × 10.9 cm, with isointense signals in both T1 (**a**) and T2 (**b**) and heterogeneous enhancement after contrast administration, with necrosis in the central area

In a routine consult, his abdominal ultrasound (US) showed a cirrhotic liver with the presence of a hypoechogenic and heterogeneous nodule in the left lobe measuring approximately 7 cm with lymph nodes on the gastrohepatic omentum, the largest measuring 24 mm. The patient was asymptomatic. His physical examination revealed only hepatomegaly without evidence of ascites. His spleen was not palpable, and his peripheral lymph nodes were not enlarged. Initial laboratory findings showed normal hemoglobin level and leukocyte count, with mild thrombocytopenia (107 × 10^3^/μl; normal range 150–350 × 10^3^/μl). His international normalized ratio was slightly elevated (1.3; normal range 0.8–1.2). His liver function test results revealed small elevations of aspartate aminotransferase (AST) (37 U/L; normal range 5–34 U/L) and bilirubin (1.56 mg/dl; normal range < 1.2 mg/dl). His albumin levels were slightly decreased (3.3 g/dl; normal range 3.4–4.8 g/dl). Serologic test results for HBV, HCV, and HIV 1 and 2 were negative. The patient’s serum α-fetoprotein (AFP), carbohydrate antigen 19-9 (CA 19-9), and carcinoembryonic antigen (CEA) were within normal range; his lactate dehydrogenase (LDH) level was normal, and his β_2_-microglobulin was slightly elevated (3.49 mg/L; normal range 0.97–2.64 mg/L). Abdominal magnetic resonance imaging (MRI) revealed a cirrhotic and enlarged liver with a voluminous nodular lesion in the left lobe measuring 15.6 × 10.9 cm, along with isointense signals in both T1 and T2, showing minimal and heterogeneous enhancement after contrast administration; it also revealed some necrosis in the center of the lesion (Fig. 1).

**Fig. 2:**
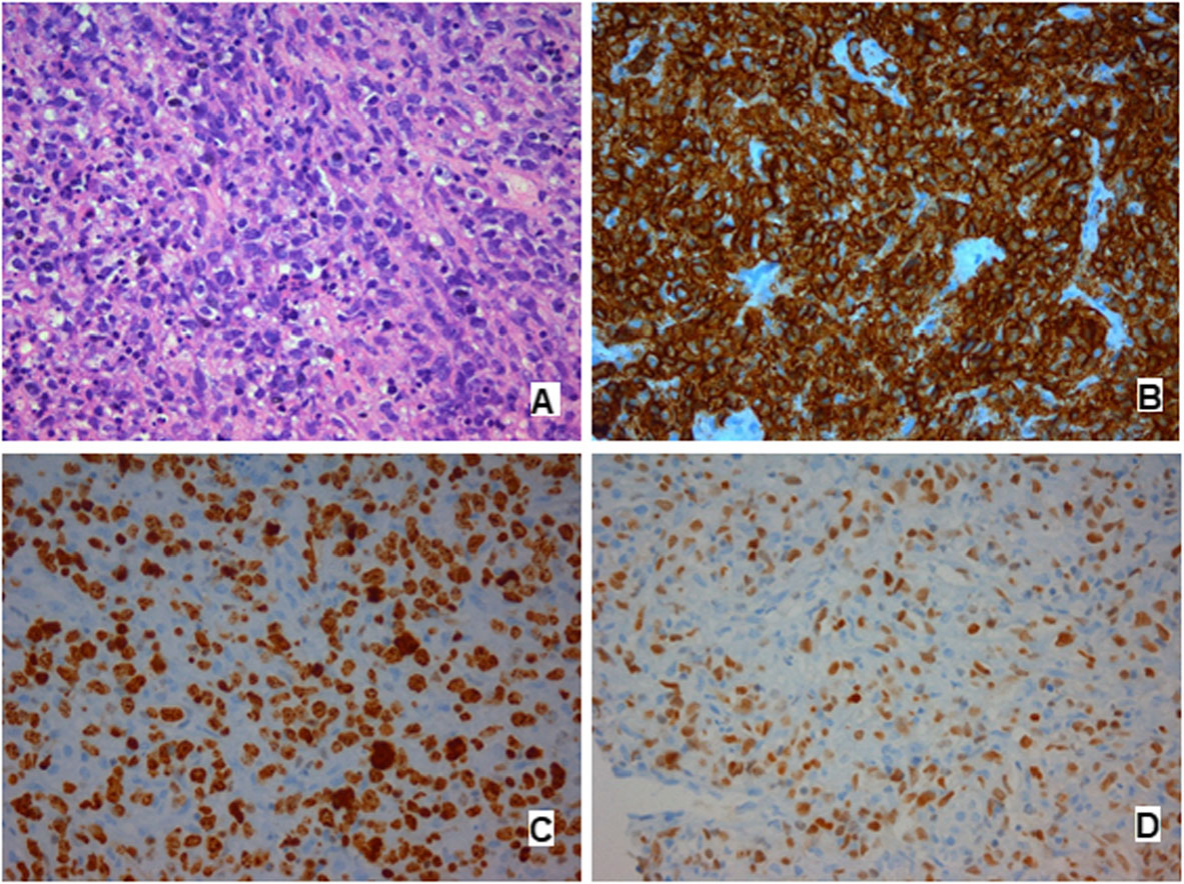
Histological and immunohistochemical staining of the liver nodule. **a** Hematoxylin and eosin stain. **b** Anti-CD20. **c** Anti-Ki67. **d** Anti-MUM1

A percutaneous liver biopsy was performed. Histological findings revealed liver tissue infiltrated by DLBCL. The results of immunohistochemical staining were positive for CD45, CD20, Bcl6, and MUM-1 and negative for CD3, CD5, CD23, CD30, and cyclin 1. The proliferation factor measured by Ki67 was > 90% (Fig. 2).

**Fig. 3:**
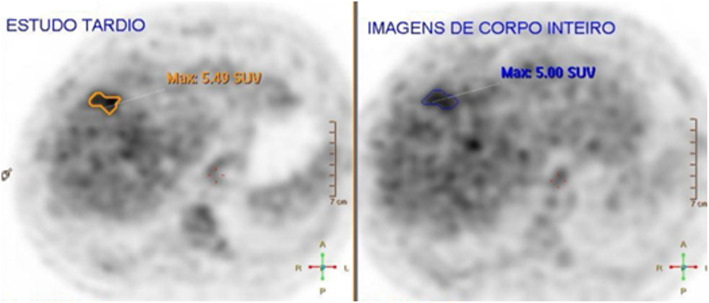
Position emission tomography/computed tomography showing irregular liver uptake and an ill-defined area with higher fluorodeoxyglucose uptake in liver segments VIII/IV, with maximal SUV=5 at initial phase (right), and SUV=5.5 at the delayed phase (left)

The patient was then referred to the hematology department for evaluation and treatment. He remained asymptomatic. His Eastern Cooperative Oncology Group performance status was 0, and the finding of his physical examination was unremarkable. His bone marrow biopsy did not reveal lymphoma infiltration. Due to limitations in access to position emission tomography (PET)/computed tomography (CT) in our institution, PET/CT could only be performed after treatment initiation (first cycle), showing irregular liver uptake, mainly in the VIII/IV liver segments (Fig. 3). A PHL diagnosis was made and classified as Ann Arbor stage IE. He was started on immunochemotherapy with R-CHOP (rituximab, cyclophosphamide, doxorubicin, vincristine, prednisone), and six treatment cycles were planned.

The patient had several toxicities secondary to treatment. He developed intestinal mucositis (grade 2) after the first cycle and hospitalization due to febrile neutropenia (grade 3) with no identifiable microbiological cause after second and third cycles. Interim PET/CT was not performed. After the fifth cycle, he was hospitalized due to pneumonia requiring mechanical ventilation, and he died despite intensive medical care.

## Discussion

First described in 1965 by Ata *et al.* [9], PHL is defined as lymphoma confined to the liver without any involvement of other organs or leukemic changes in the peripheral blood for at least 6 months after diagnosis [10]. Because the incidence of hepatic involvement in NHL ranges from 16% to 22%, careful investigation to exclude primary disease elsewhere is crucial [11]. It can occur at any age but is usually diagnosed around the fifth or sixth decade of life, with male predominance [12].

Clinical presentation is highly variable, ranging from asymptomatic to onset of fulminant hepatic failure with rapid progression to coma and death [13]. The most common presenting symptom is abdominal pain, which can occur in up to 70% of patients [14]. Other symptoms include weight loss, night sweats, fever, fatigue, anorexia, nausea, and vomiting. Hepatomegaly is the most common finding on physical examination, being present in 75–100% [15]; jaundice is found in less than 5% of patients [14]. Lei *et al.* [16] proposed the following diagnostic criteria for PHL: At presentation, the symptoms are caused mainly by liver involvement, absence of clinically palpable lymphadenopathy and no radiological evidence of distant lymphadenopathy, and absence of leukemic blood involvement in the peripheral blood smear. Laboratory findings associated with PHL include abnormal liver tests results, with nonspecific elevations of AST, alanine aminotransferase, alkaline phosphatase, and bilirubin levels [17]. LDH elevation is common, being present in 30–80% of patients [16]. Tumor markers such as AFP, CA 19-9, and CEA are within normal range in almost 100% of cases [18], helping to differentiate PHL from hepatocellular carcinoma or metastatic disease.

On the basis of liver infiltration, the most common presentation of PHL is a solitary lesion, which occurs in approximately 55–60%, followed by multiple nodular lesions in approximately 35–40% [19]. Diffuse infiltration is extremely uncommon, showing only hepatomegaly in the absence of any definite liver mass [20]; this subtype is associated with poor prognosis. Imaging features of PHL are nonspecific. On US, lesions are usually hypoechoic compared with surrounding normal liver parenchyma [21]. As seen by non-contrast-enhanced CT, lesions are typically hypodense with soft tissue attenuation. After administration of intravenous contrast, two different types of enhancement patterns can occur. First, most cases show minimal to absent enhancement on all phases, and, when enhancement is seen, it is usually less than surrounding hepatic parenchyma. Second, less commonly, enhancement of the rim of the lesion with a central nonenhancing area is seen, giving a target-like appearance to the lesion [22]. On MRI, lesions usually are hypointense to isointense on T1-weighted images and hyperintense on T2-weighted images, with minimal to absent enhancement on dynamic postgadolinium imaging [22, 23]. Due to this lack of specific clinical, laboratory, and imaging features, definitive diagnosis of PHL requires liver biopsy compatible with lymphoma in the absence of extrahepatic disease [24]. Immunohistochemical studies are essential to determine the correct subtype. If a nodular lesion is not visible on imaging for percutaneous biopsy, the transjugular approach may be a reasonable option [25].

The optimal treatment for PHL is not yet defined. Due to chemosensitivity in PHL, most patients are treated with chemotherapy. The gold standard for DLBCL is the R-CHOP regimen, in which traditional CHOP (cyclophosphamide, doxorubicin, vincristine, and prednisone) is administered with addition of rituximab (monoclonal antibody against CD20), prolonging survival with minimal toxicity [26]. In some cases, a multimodality approach including surgery and radiotherapy is also an option. The role of surgery is not fully clarified, but it may be used in patients with localized disease; there are reports that liver resection followed by adjuvant chemotherapy and/or radiotherapy is associated with a good prognosis [27].

The prognosis of PHL was considered very poor in the past, with median survival as low as 6 months; however, recent studies indicate that patients with PHL may have a more favorable prognosis than described in previous reports, with a 5-year survival rate of 77–83% [28]. Poor prognostic factors include massive liver infiltration, high proliferative index, advanced age, constitutional symptoms, bulky disease, unfavorable histologic subtype, elevated LDH levels, cirrhosis, elevated levels of β_2_-microglobulin, and comorbid illnesses [29].

## Conclusion

PHL is an extremely rare disease, presenting with nonspecific symptoms and variable laboratory and imaging findings. It should be part of the differential diagnosis in a patient presenting with nodular hepatic lesions, especially in the presence of normal tumor markers and/or elevated LDH levels. Histology is mandatory for definitive diagnosis. It is important to recognize PHL because it is a chemosensitive disorder, allowing early treatment and improved overall survival.

## Data Availability

Not applicable.
